# Immediate effects of upper cervical spinal manipulation on cervical sensorimotor control in individuals with chronic primary cervical pain: an exploratory randomized controlled trial

**DOI:** 10.1186/s12998-026-00626-2

**Published:** 2026-01-16

**Authors:** Minwoo Lee, Yongwoo Lee

**Affiliations:** Department of Physical Therapy, Sahmyook University, Seoul, 01795 Republic of Korea

**Keywords:** Neck pain, Chronic pain, Manipulation, Spinal, Atlanto-Axial joint, Psychomotor performance, Proprioception

## Abstract

**Background:**

Individuals with chronic primary cervical pain (CPCP) often exhibit cervical sensorimotor impairment. Although upper cervical spinal manipulation (UCSM) is commonly used, its immediate effects on sensorimotor control remain unclear. This study investigated the immediate effects of UCSM at the C1–C2 segment on cervical sensorimotor control in individuals with CPCP using the cervical movement sense (CMS) test.

**Methods:**

This study was an exploratory, single-blind, sham-controlled randomized controlled trial with a blinded outcome assessor. Thirty-five individuals with CPCP were recruited between May and June 2024 and randomly assigned to an experimental group (EG, *n* = 18), which received UCSM, or a control group (CG, *n* = 17), which received sham manipulation. The primary outcome was movement accuracy (MA), and secondary outcomes included movement time (MT), movement speed (MS), and movement accuracy–time ratio (MAT). These variables were assessed before and immediately after the intervention using a customized algorithm for video analysis in the CMS test. Each kinematic variable was analyzed using a two-way mixed-design ANOVA.

**Results:**

For MA, the Time × Group interaction was not statistically significant (*p* = 0.050). However, significant Time × Group interactions were observed for MT (*p* = 0.003) and MS (*p* = 0.001). Post-hoc analysis revealed that the EG showed a significant 11% decrease in MS (*p* = 0.002), whereas the CG showed a significant 13% decrease in MT (*p* = 0.009) and a significant 8% increase in MS (*p* = 0.042). No significant interaction was observed for the MAT (*p* = 0.055).

**Conclusion:**

This exploratory trial suggests that UCSM may have elicited a transient slowing of head and neck movement in the CMS test compared with sham manipulation. Under the task instructions emphasizing accuracy, this slowing may reflect a movement pattern favoring accuracy over speed, aligning with the speed–accuracy trade-off. Considering the descriptive trend toward increased MA, these kinematic changes may also be compatible with compensatory sensorimotor control immediately after UCSM. However, given the exploratory nature of this study, these interpretations should be accepted with caution. Future research is needed to validate these exploratory findings in a larger sample and to determine their long-term clinical relevance.

**Trial registration:**

This trial was retrospectively registered with the Clinical Research Information Service (CRIS), Republic of Korea (KCT00010683) on June 25, 2025.

**Supplementary Information:**

The online version contains supplementary material available at 10.1186/s12998-026-00626-2.

## Background

Neck pain is a highly prevalent musculoskeletal disorder. According to the Global Burden of Disease Study 2017, the global age-standardized point prevalence was 3551 per 100,000 population, and the incidence was 807 per 100,000 population in 2017 [1]. Approximately 50–85% of individuals with neck pain experience recurrence within one to five years [2], and the chronicity of this condition significantly interferes with occupational and daily activities, diminishing quality of life [3]. To better classify this type of persistent, non-specific pain, the International Association for the Study of Pain (IASP) has recently defined chronic primary cervical pain (CPCP) as neck pain that persists for longer than 3 months, is associated with significant emotional distress or functional disability, and is not better accounted for by another diagnosis [4].

Sensorimotor control refers to the complex central nervous system processes that integrate visual, vestibular, and proprioceptive inputs to maintain functional joint stability and coordinate appropriate motor responses [5, 6]. The cervical spine, with its high density of mechanoreceptors and extensive central and reflex connections to the visual and vestibular systems, plays a critical role in providing proprioceptive input [7]. Neck pain can disrupt these proprioceptive inputs, leading to cervical sensorimotor impairment that contributes to symptom recurrence and chronicity [8, 9]. Even after substantial neck pain reduction, impaired cervical proprioception may persist, adversely affecting long-term sensorimotor function [9, 10]. The clinical relevance of such cervical sensorimotor impairments is significant, as they are closely linked to symptoms like cervicogenic dizziness, unsteadiness, and visual disturbances, which contribute to persistent disability [5, 7–9, 11]. Consequently, addressing cervical sensorimotor impairment has become a key aspect of CPCP management [7, 12].

The cervical movement sense (CMS) test has emerged as a clinically feasible tool to quantify these impairments [13–15]. This test requires participants to trace a predefined pattern by moving their head and neck, a task that assesses the ability to integrate visual and proprioceptive feedback for accurate motor control, thereby reflecting visuomotor performance as an indicator of cervical sensorimotor control [15, 16]. Kinematic variables derived from the CMS test, such as the accuracy, time, and speed of movement, provide insights into motor control strategy and execution [13–17].

Importantly, these variables may be associated with the speed–accuracy trade-off, a fundamental principle in motor control that describes an inverse relationship between movement speed and accuracy [18, 19]. Previous studies using the CMS test have shown that individuals with chronic neck pain have demonstrated greater errors, increased movement time [14, 17], and reduced “NormAcuity” (i.e., acuity normalized by time), reflecting a speed–accuracy trade-off when compared to healthy controls [13]. However, most previous studies have been observational [13, 14, 17], and few have quantitatively examined the effects of therapeutic interventions using the CMS test [16, 20]. To elucidate the underlying mechanisms of change, intervention studies need to dissect the fundamental components of cervical sensorimotor control rather than relying solely on composite variables.

Spinal manipulation, a manual therapy technique involving high-velocity, low-amplitude thrusts, is commonly used to manage musculoskeletal pain and restore function [21–24]. It has been proposed that spinal manipulation can modulate proprioceptive input from mechanoreceptors such as muscle spindles [25, 26], as well as peripheral nociceptive input and central pain processing [24]. These effects may enhance somatosensory processing, contribute to sensorimotor integration, and thereby facilitate neuromuscular responses [21].

Additionally, spinal manipulation is often applied to specific spinal segments identified with joint dysfunction, based on the rationale that its effects may vary according to the neuroanatomical characteristics of the targeted segments [27, 28]. However, this concept of segmental specificity is a topic of ongoing debate [27–32]. While some neurophysiological evidence supports segment-specific effects [27, 28], recent evidence suggests that targeting a specific spinal segment may be less critical for certain clinical outcomes like pain [29–32].

The upper cervical spine, particularly the C1–C2 segment, plays a crucial role in proprioceptive processing. Its high density of muscle spindles in the deep suboccipital muscles and joint mechanoreceptors provide finely tuned proprioceptive feedback essential for precise head movement control [9, 33, 34]. Dysfunction in the upper cervical region, originating from muscles or ligaments, may disrupt cervical proprioceptive signaling [35, 36], potentially contributing to sensorimotor impairment such as head and eye movement and postural stability [5, 7]. However, despite the clinical relevance of this region, the effects of spinal manipulation at the C1–C2 segment on cervical sensorimotor control, as assessed by the CMS test, have not yet been clearly established.

Therefore, this exploratory study aimed to investigate the immediate effects of upper cervical spinal manipulation (UCSM) applied to the C1–C2 segment on cervical sensorimotor control in individuals with CPCP, as assessed by the CMS test.

## Methods

### Study design

**Fig. 1:**
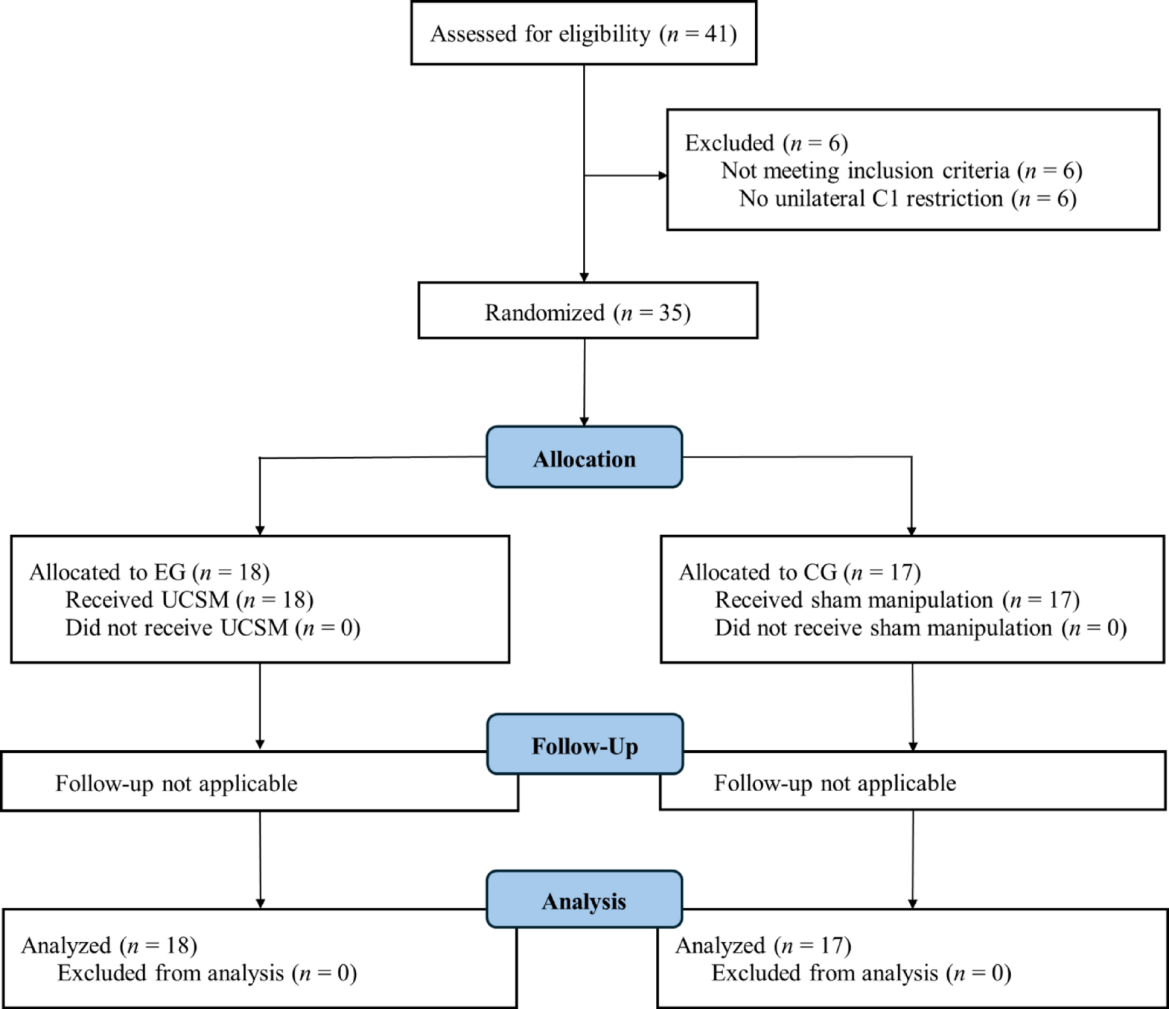
CONSORT flow diagram

This study was an exploratory, single-blind, sham-controlled randomized controlled trial with blinded outcome assessment, in which participants were randomly assigned to either an experimental group (EG) or a control group (CG). Randomization and reporting followed Consolidated Standards of Reporting Trials (CONSORT) guidelines [37] (Fig. 1). The study was conducted in accordance with the ethical principles outlined in the Declaration of Helsinki. Ethical approval was obtained from the Institutional Review Board (IRB) of Sahmyook University (approval number: SYU 2023-11-001-002). The trial was also retrospectively registered with the Clinical Research Information Service (CRIS), Republic of Korea (registration number: KCT00010683; https://cris.nih.go.kr/cris/search/detailSearch.do? seq=30414) on June 25, 2025. The trial was retrospectively registered at a public registry after recruitment had begun, which may increase the perceived risk of reporting bias, although the statistical analysis plan was defined a priori in the IRB-approved protocol.

### Participants

Thirty-five participants with CPCP were recruited via social media advertisements and bulletin boards at S University and B Orthopedic Clinic located in Seoul, Republic of Korea between May and June 2024. All participants received information about the assessment procedures and objectives and provided written informed consent. Participant eligibility was determined through a written screening questionnaire, based on self-reported medical history, supplemented by a physical examination. No imaging examinations were performed.

The inclusion criteria were: (1) age between 20 and 50 years to minimize confounding from age-related degenerative changes common in older populations [38]; (2) neck pain lasting more than 3 months; (3) pain on palpation of the cervical muscles or during neck movement in the posterior region from C0 to T4 [39]; (4) a visual analogue scale (VAS) score ≥ 20 mm to ensure at least a “mild” level of pain intensity, as defined by the IASP for the ICD-11 classification [40]; (5) unilateral restricted mobility at the C1 segment, determined by manual palpation [41].

Based on previous studies [42, 43], participants were excluded if they met any of the following criteria: (1) fear or strong apprehension regarding spinal manipulation; (2) history of cervical spine surgery; (3) self-reported physician diagnosis of any of the following conditions: congenital malformation of the head or cervical spine, rheumatoid disease, spinal osteoporosis or osteopenia, ankylosing spondylitis, or malignancy; (4) traumatic cervical spine injury within the previous year (e.g., fracture, sprain, whiplash); or (5) current use of muscle relaxants, analgesics, anti-inflammatory drugs, or antidepressants.

### Randomization

Participants were randomly assigned to the EG or CG using block randomization (block size = 4), generated by Random Allocation Software (version 2.0, Isfahan University, Iran). Allocation concealment was maintained using sequentially numbered, opaque, sealed envelopes prepared by an independent researcher not involved in recruitment or outcome assessment.

### Blinding

This study was a single-blind trial where participants were blinded to their group allocation. To minimize potential detection bias, all outcome measurements were conducted by a single, separate assessor who was also blinded to the participants’ group assignment. Participants were told that they would be randomly assigned to one of two intervention groups, both described as manual procedures. They were also informed that cavitation sounds might or might not occur during treatment, regardless of group allocation. To enhance blinding integrity, both procedures were performed in the same treatment room, with identical participant positioning, and for the same duration. The term “sham manipulation” was not used during participant information to minimize expectancy bias. The success of participant blinding was not evaluated, which is a limitation of the study.

### Interventions

Both interventions targeted the C1–C2 articulation of the upper cervical spine. The EG received UCSM, while the CG received sham manipulation. All interventions were administered by a physical therapist licensed in the Republic of Korea with over five years of clinical experience in performing spinal manipulation. Although the therapist did not hold a specific post-graduate certification, their competency was ensured by their professional license and extensive clinical experience. The intervention was applied unilaterally to the side with restricted C1 mobility, as determined by manual palpation [41, 44].

### UCSM

**Fig. 2:**
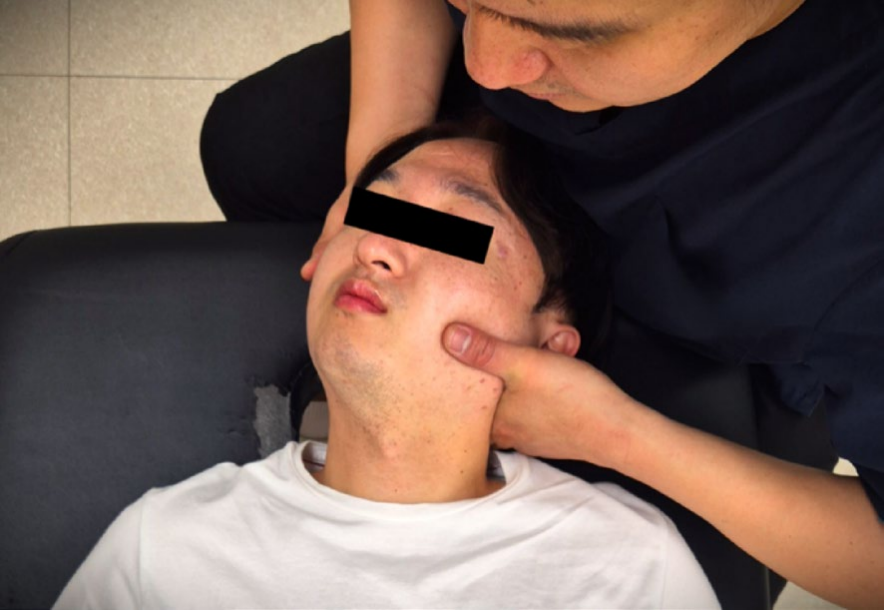
Upper cervical spinal manipulation (UCSM) targeted the C1–C2 articulation

The UCSM was applied unilaterally to the side with restricted C1 mobility, as determined by manual palpation [41]. The following describes the procedure for a left-sided restriction. Participants were positioned supine on the treatment table. The examiner placed the lateral aspect of the left index finger on the participant’s left posterolateral C1 posterior arch. Supporting the participant’s head with the right hand, the examiner passively moved the neck into left lateral flexion and right rotation until the end range of tissue tension was felt. After instructing the participant to “take a deep breath”, a high-velocity, low-amplitude thrust was delivered in an arc toward the underside of the right eye (a superior-medial direction). A maximum of two attempts was allowed per participant [45]. The right side was treated in the same manner (Fig. 2).

### Sham manipulation

Participants were positioned supine on the treatment table. The examiner placed the left index finger on the left posterolateral C1 posterior arch and, after instructing the participant to take a deep breath, performed a thrust-like maneuver toward the underside of the right eye. No actual thrust force or passive joint movement was applied [45–47].

### Assessment procedure

**Fig. 3:**
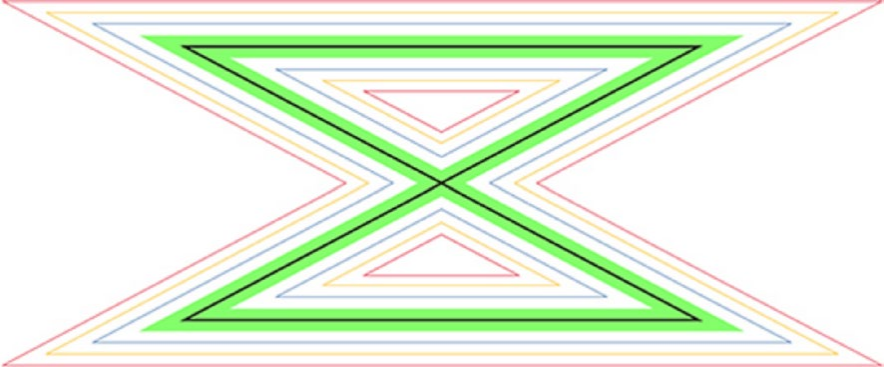
Zigzag pattern used in the cervical movement sense (CMS) test was printed on A3 paper (1 mm black line; 234 mm horizontal, 266 mm diagonal; total length, 1000 mm)

**Fig. 4:**
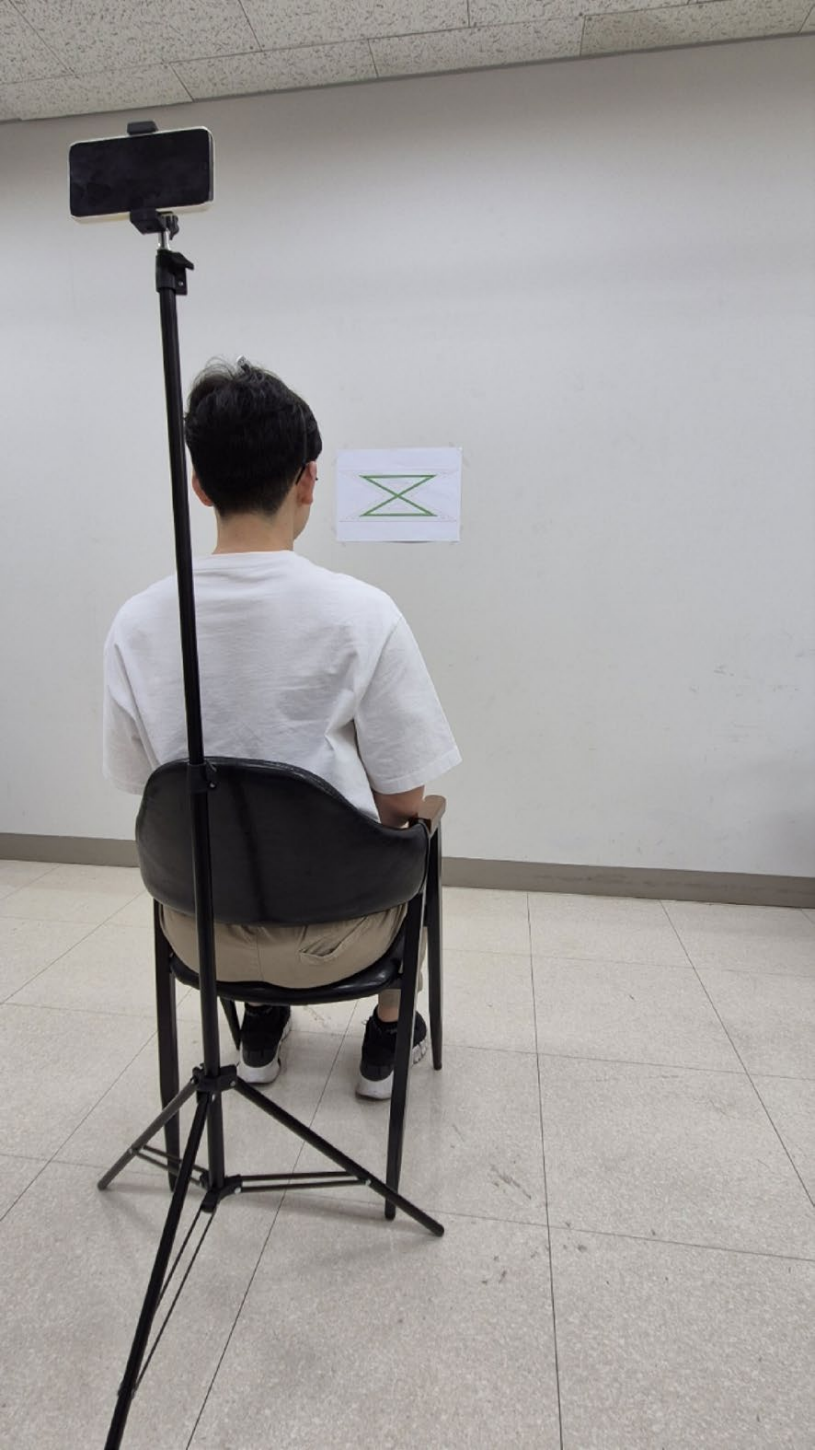
A participant wore a headband with a laser pointer and was seated on a chair positioned 1000 mm away, facing the wall

For the CMS test, participants wore a headband with a laser pointer and sat on a chair facing the wall. A zigzag pattern printed on A3 paper (1 mm black line, 234 mm horizontal, 266 mm diagonal, total length 1000 mm) was affixed to the wall at eye level, 1000 mm from the participant [15] (Fig. 3). Video was recorded from behind using a tripod-mounted smartphone (iPhone 12 Pro Max, Apple Inc., USA, 2020) (Fig. 4).

Participants were instructed to trace the black line in the zigzag pattern as accurately as possible at a self-chosen speed by moving their head and neck. While the basic procedure was based on Röijezon et al. [13], the movement directions and trial structure were adapted to fit the design of the present study.

Each trial began with the laser dot positioned at one of the four reference corners of the zigzag pattern, initiated by a signal from the examiner. Participants were instructed to hold the laser dot stationary at the start point for approximately one second before initiating the movement, and to do the same upon returning to the start point at the end of the trial. The trials were conducted in the following directions: upper left to lower right, upper right to lower left, lower left to upper right, and lower right to upper left, thereby completing a full circuit by returning to the start point. Each trial was recorded in .mp4 format (1280 × 720 resolution, 30 fps), with four trials conducted both pre- and post-intervention.

### Video analysis

For the video analysis, data from the four trials conducted at each time point, pre- and post-intervention, were averaged for each movement direction. Video analysis was conducted using a customized algorithm in MATLAB, based on the methodology described by Röijezon et al. [13, 48]. Although the original source code was not utilized, the algorithm and procedures were independently implemented using similar image processing techniques reported in previous studies [13, 48]. Whereas the original approach fully automated all processing steps, the present method was modified to allow manual selection of the start and end frames, thereby enhancing precision and user control. The key steps included: (1) extracting the black line and detecting the four reference corners in the zigzag pattern, (2) extracting the laser dot and normalizing its size, (3) calculating and preprocessing the frame-by-frame pixel distance from the start point, (4) visualizing the pixel distance and manually selecting the start and end frames of laser dot movement, (5) determining laser dot contact with the black line, and (6) calculating the CMS kinematic variables (**see Supplementary Material 1**).

### Outcome measures

From the video analysis described above, four kinematic variables were calculated. The primary outcome was the movement accuracy (MA). MA was defined as the percentage of laser dot contact with the black line in the zigzag pattern. It represents a key aspect of cervical sensorimotor control, reflecting visuomotor performance by integrating proprioceptive and visual information [16]. Secondary outcomes included movement time (MT), movement speed (MS), and movement accuracy-time ratio (MAT). MT was defined as the time required to complete the task. MS was calculated as the total distance traveled by the laser dot divided by MT, representing the average movement speed [13, 15]. MAT was defined as MA normalized by MT, reflecting the speed–accuracy trade-off.

Röijezon et al. [13] assessed the test–retest reliability of the CMS test with the zigzag pattern using custom-made software with an automated video-based scoring system. They reported intraclass correlation coefficients (ICCs) of 0.84 for “Acuity”, 0.93 for “Time”, 0.96 for “Speed”, and 0.91 for “NormAcuity”.

### Sample size calculation

Although this study was exploratory, an a priori power analysis was conducted to ensure scientific rigor and estimate a conservative sample size using G*Power (version 3.1.9.4, Germany, 2019). Given the lack of previous studies reporting CMS test outcomes following comparable interventions, a medium effect size (Cohen’s *d* = 0.50, equivalent to *f* = 0.25) [49] was assumed for a repeated measures analysis of variance (ANOVA, within-between interaction). With a significance level (*α*) of 0.05, power (1 − *β*) of 0.80, correlation among repeated measures estimated at 0.50, and two groups (EG and CG) measured at two time points (pre- and post-intervention), the calculation indicated that 34 participants were required. A total of 35 participants were ultimately recruited for this study, thereby meeting this requirement.

### Statistical analysis

Statistical analysis was performed using SPSS (version 22.0; IBM Corp., Armonk, NY, USA). The normality of the raw data was assessed using the Shapiro–Wilk test. All four CMS kinematic variables of the raw data showed a non-normal distribution, characterized by a right skewness; therefore, a natural logarithm (ln) transformation was applied [50]. After this transformation, the key assumptions for the ANOVA were confirmed to be met: the normality of the residuals was verified using the Shapiro–Wilk test, and the homogeneity of variances of the residuals was verified using Levene’s test. After confirming these assumptions, two-way mixed-design ANOVAs were conducted for each CMS kinematic variable (MA, MT, MS, and MAT), with Time (pre- and post-intervention) as the within-subject factor and Group (EG and CG) as the between-subjects factor. When the interaction effect was significant, Bonferroni-adjusted post-hoc within-group comparisons were conducted to compare pre- and post-intervention values within each group. For interactions that did not reach statistical significance, exploratory within-group comparisons were also performed to examine potential trends. For all log-transformed CMS kinematic variables, the results of the within-group comparisons were reported as geometric mean ratios and percentage changes based on back-transformed log values. Effect sizes were reported as partial eta squared (*η²*_p_), with 0.01 considered small, 0.06 medium, and 0.14 large [49]. Statistical significance level (*α*) was set at 0.05.

## Results

A total of 41 participants with CPCP were screened for eligibility. Six participants were excluded as they did not present with unilateral C1 restriction. The final sample consisted of 26 men and 9 women, with mean age of 28.03 ± 5.69 years, mean height of 173.40 ± 8.19 cm, mean weight of 72.31 ± 16.02 kg, mean body mass index (BMI) of 23.89 ± 3.91 kg/m², mean VAS score of 44.60 ± 14.30 mm, and mean pain duration of 10.83 ± 11.38 months. No adverse events were reported following the interventions. Baseline demographic characteristics and CMS kinematic variables by group are presented in Table 1. Descriptive statistics for CMS kinematic variables at pre- and post-intervention, along with the results of the two-way mixed-design ANOVA, are summarized in Table 2.

**Table 1:** Baseline demographic characteristics and CMS kinematic variables (*n* = 35)

	EG (*n* = 18)	CG (*n* = 17)
Demographic characteristics		
Gender (male / female)	13 / 5	13 / 4
Age (years)	26.50 ± 3.17	29.65 ± 7.25
Height (cm)	172.83 ± 9.22	174.00 ± 7.18
Weight (kg)	70.67 ± 14.48	74.06 ± 17.79
BMI (kg/m²)	23.52 ± 3.56	24.28 ± 4.34
VAS score (mm)	42.72 ± 16.08	46.59 ± 12.31
Pain duration (months)	11.28 ± 10.09	10.35 ± 13.27
CMS kinematic variables^a^		
MA (%)	30.73 [27.74, 34.91]	32.58 [26.53, 40.62]
MT (s)	26.04 [15.94, 32.31]	28.10 [21.73, 42.06]
MS (mm/s)	53.15 [41.55, 73.13]	48.20 [36.95, 58.30]
MAT	1.39 [1.08, 1.66]	1.15 [0.75, 1.84]

Values are presented as mean ± standard deviation (SD) for normally distributed data.

^a^Presented as median [interquartile range, IQR] due to non-normal distribution.

EG: experimental group; CG: control group; BMI: body mass index; VAS: visual analogue scale; MA: movement accuracy; MT: movement time; MS: movement speed; MAT: movement accuracy–time ratio.

**Table 2:** Descriptive statistics and two-way mixed-design ANOVA results for CMS kinematic variables (*n* = 35)

CMS kinematic variables	Group	Pre-intervention	Post-intervention	Time × Group			Within-group changes
				*F*	*p*	*η* ^2^_p_	
MA (%)	EG	30.73 [27.74, 34.91]	32.06 [30.40, 37.37]	4.13	0.050^a^	0.11	1.08 (1.03 to 1.13)^b^
	CG	32.58 [26.53, 40.62]	30.50 [25.55, 40.91]				1.00 (0.95 to 1.05)^b^
MT (s)	EG	26.04 [15.94, 32.31]	30.24 [18.71, 36.93]	10.15	0.003	0.24	1.08 (0.99 to 1.19)
	CG	28.10 [21.73, 42.06]	25.64 [19.56, 31.28]				0.87 (0.79 to 0.97)^**^
MS (mm/s)	EG	53.15 [41.55, 73.13]	44.05 [38.55, 61.63]	14.75	0.001	0.31	0.89 (0.83 to 0.96)^**^
	CG	48.20 [36.95, 58.30]	50.10 [42.45, 63.40]				1.08 (1.00 to 1.16)^*^
MAT	EG	1.39 [1.08, 1.66]	1.16 [0.86, 1.78]	3.96	0.055	0.11	0.99 (0.89 to 1.10)^b^
	CG	1.15 [0.75, 1.84]	1.23 [1.00, 1.81]				1.15 (1.03 to 1.28)^b^

Pre- and post-intervention values are presented as median [IQR] of raw data.

Within-group changes are presented as geometric mean ratios of post- to pre-intervention values (95% confidence interval, CI), based on back-transformed log values.

^a^Not statistically significant (exact *p* = 0.0504).

^b^Presented exploratively to examine potential trends, as the Time × Group interaction was not statistically significant.

EG: experimental group; CG: control group; MA: movement accuracy; MT: movement time; MS: movement speed; MAT: movement accuracy–time ratio.

^*^p < 0.05; ^**^p < 0.01 for Bonferroni-adjusted post-hoc within-group comparisons.

For log-transformed MA, the Time × Group interaction effect was not statistically significant (*F*(1, 33) = 4.13; *p* = 0.050; *η²*_p_ = 0.11). However, a significant main effect of Time was found (*F*(1, 33) = 4.96; *p* = 0.033; *η²*_p_ = 0.13), indicating an overall 4% improvement in MA from pre- to post-intervention (geometric mean ratio = 1.04; 95% CI: 1.00 –1.08). The main effect of Group was not significant (*F*(1, 33) = 0.00; *p* = 0.963; *η²*_p_ = 0.00).

For log-transformed MT, a significant Time × Group interaction effect was found (*F*(1, 33) = 10.15; *p* = 0.003; *η²*_p_ = 0.24). Bonferroni-adjusted post-hoc within-group comparisons, based on back-transformed log values, revealed no significant change in the EG (geometric mean ratio = 1.08; 95% CI: 0.99 to 1.19; *p* = 0.094), whereas the CG showed a significant 13% decrease (geometric mean ratio = 0.87; 95% CI: 0.79 to 0.97; *p* = 0.009). No significant main effects of Time (*F*(1, 33) = 0.61; *p* = 0.439; *η²*_p_ = 0.02) or Group (*F*(1, 33) = 0.16; *p* = 0.693; *η²*_p_ = 0.01) were observed.

For log-transformed MS, a significant Time × Group interaction effect was also found (*F*(1, 33) = 14.75; *p* = 0.001; *η²*_p_ = 0.31). Bonferroni-adjusted post-hoc within-group comparisons, based on back-transformed log values, showed a significant 11% decrease in the EG (geometric mean ratio = 0.89; 95% CI: 0.83 to 0.96; *p* = 0.002), and a significant 8% increase was observed in the CG (geometric mean ratio = 1.08; 95% CI: 1.00 to 1.16; *p* = 0.042). No significant main effects of Time (*F*(1, 33) = 0.64; *p* = 0.430; *η²*_p_ = 0.02) or Group (*F*(1, 33) = 0.16; *p* = 0.693; *η²*_p_ = 0.01) were observed.

**Fig. 5:**
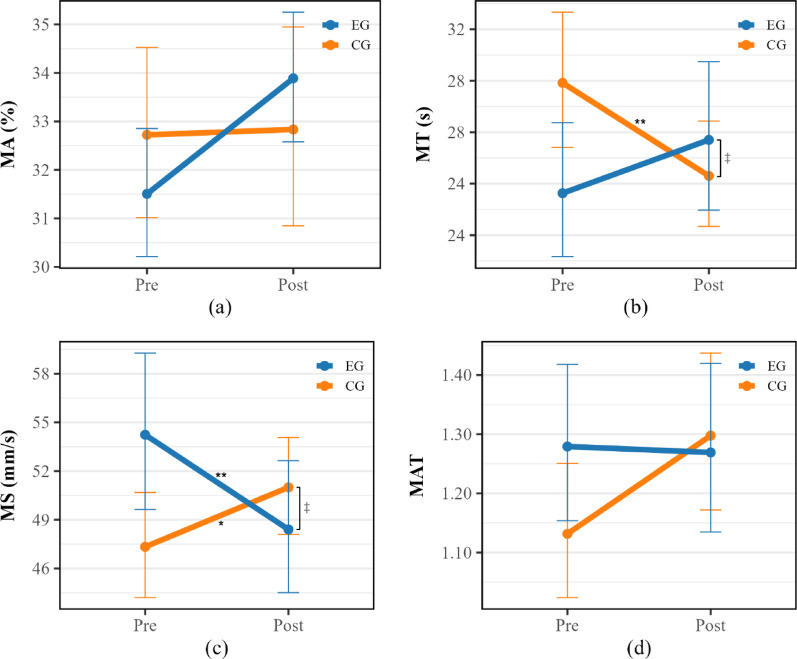
Results of two-way mixed-design ANOVA for CMS kinematic variables, showing** a** movement accuracy (MA), **b** movement time (MT), **c** movement speed (MS), and** d** movement accuracy–time ratio (MAT). Each data point represents the geometric mean, and error bars indicate the standard error (SE), with all values back-transformed from the log scale. Significant Time × Group interaction effects are indicated to the right of the data points (^‡^*p* < 0.01). For variables with a significant interaction, Bonferroni-adjusted post-hoc within-group comparisons are indicated adjacent to the corresponding group lines (^*^p < 0.05; ^**^p < 0.01)

For log-transformed MAT, the Time × Group interaction effect was not statistically significant (*F*(1, 33) = 3.96; *p* = 0.055; *η²*_p_ = 0.11). No significant main effects of Time (*F*(1, 33) = 3.15; *p* = 0.085; *η²*_p_ = 0.09) or Group (*F*(1, 33) = 0.12; *p* = 0.729; *η²*_p_ = 0.00) were found (Table 2; Fig. 5).

## Discussion

This study investigated the immediate effects of UCSM applied to the C1–C2 segment on cervical sensorimotor control in individuals with CPCP, using the CMS test. Immediately after the intervention, the two groups exhibited different kinematic patterns. The Time × Group interaction was not significant for MA, whereas significant Time × Group interactions were observed for MT and MS. Specifically, the EG demonstrated a significant decrease in MS, whereas the CG showed a significant decrease in MT and a significant increase in MS. For MAT, the Time × Group interaction was not statistically significant.

Both the EG and the CG received the same task instructions to perform the CMS test “as accurately as possible at a self-chosen speed,” following the protocol of Röijezon et al. [13]. This instruction was designed to maximize accuracy without imposing explicit constraints on task performance time or speed. Therefore, the kinematic patterns observed in the present study should be interpreted within this context. Under these task instructions, the Time × Group interaction for MA was not statistically significant (*p* = 0.050; exact *p* = 0.0504). This finding aligns with the results of Miranda et al. (2016) [51], who reported no significant difference in joint position error (JPE) between cervical manipulation and sham manipulation in patients with chronic neck pain. They suggested that, even when cervical sensorimotor dysfunction is present, information from other sensory systems such as the vestibular system or the cerebellum may compensate via adaptive reweighting. Given that the CMS test was developed based on JPE [13], it is possible that MA in this study was also influenced by such adaptive reweighting, which may have attenuated between-group differences.

Although the Time × Group interaction for MA was not statistically significant, the EG showed a descriptive trend toward an approximately 8% increase in MA, whereas the CG exhibited negligible change. Given that the task instructions emphasized accuracy, this descriptive trend is compatible with an accuracy-oriented movement pattern. This possibility should be interpreted cautiously at an exploratory level.

Notably, a significant decrease in MS was observed in the EG immediately after UCSM. This finding contrasts with previous studies [52–55], which reported that cervical manipulation or passive mobilization could contribute to reduced task performance time and increased movement speed. This discrepancy may be attributed to methodological differences, including the types of visuomotor tasks used, and particularly the task instructions. Whereas those studies emphasized both accuracy and speed [52–55], the task instructions for the CMS test in this study were designed to prioritize accuracy. Under these task conditions, the EG may have exhibited a movement pattern that favored accuracy over speed.

In this context, the speed-accuracy trade-off may provide an important interpretive framework [18, 19]. The significant decrease in MS observed in the EG may suggest a movement pattern in which speed was modulated to prioritize accuracy under the given task instructions. This interpretation is compatible with the descriptive trend toward an increase in MA in the EG. At the same time, from a neurophysiological perspective, it can be speculated that these kinematic changes may reflect compensatory sensorimotor control while integrating potentially altered proprioceptive inputs immediately after UCSM. These interpretations can be discussed in relation to motor learning frameworks, in which goal-based strategic processes (explicit) and sensory prediction error–based adaptation (implicit) can operate in parallel [56, 57].

Specifically, these perspectives align with the findings of Robinault et al. [58]. They observed a decrease in muscle conduction velocity during low-intensity muscle contractions following spinal manipulation, attributing this to increased recruitment of low-threshold motor units suitable for precise motor control. Furthermore, previous studies [59, 60] on motor learning in accuracy-oriented tasks have proposed specific sensorimotor adaptation mechanisms to maintain accuracy. Franklin et al. [59] suggested that sensorimotor adaptation involves a process of increasing visuomotor feedback gains to reduce movement variability and enhance accuracy. In addition, Calalo et al. [60] reported that the sensorimotor system employs impedance control strategies, such as increased muscle co-contraction, to regulate trajectory variability and achieve accuracy. Taken together, the slowing observed in the EG could be interpreted as a subtle modulation of sensorimotor control to integrate potentially altered proprioceptive inputs, which may contribute to fine motor control. However, given the exploratory nature of the present study, these speculations should be interpreted with considerable caution.

Although the Time × Group interaction for MAT was not statistically significant, the descriptive statistics may offer exploratory insights into how each group reflected the speed-accuracy trade-off [18, 19]. In the EG, both MA and MT showed descriptive trends toward increases. In the calculation of MAT, these changes numerically offset each other, resulting in a negligible change of less than 1%. Considering the speed-accuracy trade-off, these concurrent increasing trends in both task performance time and accuracy may reflect the accuracy-oriented movement pattern discussed earlier. Nevertheless, this interpretation should be accepted with caution at an exploratory level.

In contrast, the CG exhibited a significant decrease in MT and a significant increase in MS without a significant change in MA. This pattern may reflect typical motor learning effects arising from task repetition, which are often associated with error-based motor adaptation [56, 61]. It is plausible that the numerous subtle positional adjustments required while tracing the zigzag pattern generated repetitive sensory prediction errors, which in turn progressively reduced unnecessary motor variability and updated the internal model [62, 63]. However, although both groups performed the identical repetitive task during the CMS test, the EG did not exhibit the typical pattern of increased speed (i.e., decreased MT or increased MS) as observed in the CG. This suggests that the EG exhibited a movement pattern distinct from the typical motor learning effects associated with task repetition. This discrepancy implies that the two groups may have modulated the speed-accuracy trade-off differently under the task instructions emphasizing accuracy.

Admittedly, it cannot be definitively concluded that the sham manipulation applied to the CG was physiologically entirely inert [64, 65]. It is possible that minor proprioceptive inputs from hands-on contact or non-specific effects, such as expectation or placebo effects, may have been present [65]. However, even if such effects were present, they did not appear to alter the overall pattern of increased speed in the CG. This suggests the possibility that the sham manipulation was not substantial enough to override the typical motor learning pattern, or that it elicited a different pattern of change compared to the EG receiving UCSM.

It is also important to consider whether the results observed in the EG are attributable to the specific stimulation of the C1–C2 segment. Recent studies [29–32] suggest that the specific target site of spinal manipulation may be less critical for improving clinical outcomes such as pain or disability. However, regarding neurophysiological responses, the potential for segment specificity warrants consideration [27, 28]. For instance, animal studies by Reed et al. [27] observed immediate changes in muscle spindle activity specifically at the segment where spinal manipulation was applied, suggesting a localized alteration in sensory input. Furthermore, Niazi et al. [28] applied spinal manipulation to preidentified dysfunctional upper cervical segments (C1–C3) and observed greater changes in the N30 somatosensory evoked potentials (SEPs) amplitude compared with manipulation of non-dysfunctional segments. This potential for segment specificity is further supported by the functional role of the upper cervical musculature, as prior studies [34, 66] have indicated that deep suboccipital muscles, such as the obliquus capitis inferior, function primarily as proprioceptive sensors for precise head control rather than as torque generators. Considering this proprioceptive richness [9, 33, 34], these findings suggest that stimulation applied to a specific segment may differentially modulate sensorimotor processing. However, as the present study did not directly compare different target segments, this hypothesis remains speculative and requires further clarification through future research.

The exact underlying mechanisms regarding how spinal manipulation affects sensorimotor control remain unclear. However, several neurophysiological possibilities have been proposed [21, 25, 26]. First, high-velocity, low-amplitude stimuli may alter proprioceptive inputs originating from muscle spindles and joint mechanoreceptors. Second, these changes in peripheral sensory input may influence the modulation of spinal reflex circuits. Finally, there is evidence suggesting that these alterations may also affect sensorimotor integration processes at supraspinal and cortical levels. These neurophysiological responses may offer a potential explanation for the kinematic changes observed in the EG.

Collectively, the transient slowing of movement observed in the EG may reflect a movement pattern favoring accuracy over speed under the task instructions emphasizing accuracy. From a clinical perspective, it may be reasonable to initially prioritize accuracy-oriented sensorimotor training following UCSM, before progressing to rapid or dynamic movement tasks. This approach could provide patients with an opportunity to recalibrate their sensorimotor system while adapting to potentially altered sensory inputs. However, given the exploratory nature of this study, these clinical implications should be interpreted with caution, and clinicians should closely monitor individual patient responses.

This study has several limitations that should be considered. First, the trial was registered retrospectively, which may increase the perceived risk of reporting bias, although the statistical analysis plan was defined a priori in the IRB-approved protocol. Second, it focused solely on the immediate effects of UCSM and sham manipulation, without assessing the persistence or clinical relevance of the observed effects over the medium- to long-term. Third, the generalizability of the findings may be limited by the relatively small sample size and the inclusion of a specific subgroup with unilateral C1 restriction. Fourth, participant blinding was not formally verified. Fifth, although the customized algorithm was developed based on previous studies [13, 48], its reliability and measurement error have not yet been formally evaluated. Sixth, although parametric tests were used in this study, alternative non-parametric approaches were not explored and could be considered in future research. Seventh, reliance on clinical screening without imaging means that undiagnosed structural or systemic variations in participants cannot be fully ruled out. Finally, although an a priori power analysis was performed, this study was fundamentally exploratory. Therefore, readers should be aware that these findings are preliminary and the possibility of spurious results cannot be completely excluded.

Importantly, the results support the feasibility of the experimental protocol, as evidenced by the absence of reported adverse events and the successful implementation of the CMS test. This suggests that the intervention and assessment procedures are safe and clinically feasible. Future studies should further investigate the underlying mechanisms and long-term effects using larger sample sizes and task conditions that emphasize both accuracy and speed.

## Conclusion

To our knowledge, this is the first study to assess cervical sensorimotor control using the CMS test following high-velocity, low-amplitude spinal manipulation in individuals with CPCP. This exploratory randomized controlled trial showed that UCSM and sham manipulation elicited different kinematic patterns of head and neck movement in the CMS test. Under the task instructions emphasizing accuracy, the transient slowing observed in the EG may reflect a movement pattern favoring accuracy over speed, aligning with the speed-accuracy trade-off. Considering the descriptive trend toward increased MA, this slowing may also be compatible with compensatory sensorimotor control immediately after UCSM. In contrast, the CG showed relatively faster movement, which may reflect a typical motor learning effect arising from task repetition. However, given the exploratory nature of this study, these interpretations should be accepted with caution. Importantly, the absence of reported adverse events supports the feasibility and safety of the experimental protocol. Future research is needed to validate these exploratory findings in a larger sample and to determine their long-term clinical relevance.

## Supplementary Information

Below is the link to the electronic supplementary material.

Supplementary Material 1

Supplementary Material 2

Supplementary Material 3

## Data Availability

The datasets used and/or analyzed during the current study are available from the corresponding author on reasonable request. The customized MATLAB algorithm for video analysis used in this study is provided in Additional file 1. Upon acceptance of the manuscript, this codes will be archived in Zenodo and assigned a DOI, which will be included in Additional file 1 to ensure permanent access.

## Abbreviations

CPCP: Chronic primary cervical pain
UCSM: Upper cervical spinal manipulation
CMS: Cervical movement sense
EG: Experimental group
CG: Control group
IASP: International association for the study of pain
MA: Movement accuracy
MT: Movement time
MS: Movement speed
MAT: Movement accuracy–time ratio
CONSORT: Consolidated standards of reporting trials
CRIS: Clinical research information service
IRB: Institutional review board
VAS: Visual analogue scale
fps: Frames per second
ICCs: Intraclass correlation coefficients
ANOVA: Analysis of variance
ln: Natural logarithm
BMI: Body mass index
SD: Standard deviation
IQR: Interquartile range
CI: Confidence interval
JPE: Joint position error
SEPs: Somatosensory evoked potentials

## References

[1] Safiri S, Kolahi A-A, Hoy D, Buchbinder R, Mansournia MA, Bettampadi D et al. Global, regional, and National burden of neck pain in the general population, 1990–2017: systematic analysis of the global burden of disease study 2017. BMJ. 2020;368:m791 . 10.1136/bmj.m791 PMC7249252 32217608

[2] Haldeman S, Carroll L, Cassidy JD. Findings from the bone and joint decade 2000 to 2010 task force on neck pain and its associated disorders. J Occup Environ Med. 2010;52(4):424–7. 20357682 10.1097/JOM.0b013e3181d44f3b

[3] Vernon H, Humphreys BK. Chronic mechanical neck pain in adults treated by manual therapy: a systematic review of change scores in randomized controlled trials of a single session. J Man Manipulative Ther. 2008;16(2):E42–52. 10.1179/jmt.2008.16.2.42E. 10.1179/jmt.2008.16.2.42E PMC2565115 19119388

[4] Nicholas M, Vlaeyen J, Rief W, Barke A, Aziz Q, Benoliel R, et al. The IASP classification of chronic pain for ICD-11: chronic primary pain. Pain. 2019;160:28. 10.1097/j.pain.0000000000001390. 30586068 10.1097/j.pain.0000000000001390

[5] Treleaven J. Dizziness, unsteadiness, visual disturbances, and sensorimotor control in traumatic neck pain. J Orthop Sports Phys Therapy. 2017;47(7):492–502. 10.2519/jospt.2017.7052 28622488

[6] Riemann BL, Lephart SM. The sensorimotor system, part I: the physiologic basis of functional joint stability. J Athl Train. 2002;37(1):71–9. 16558670 PMC164311

[7] Treleaven J. Sensorimotor disturbances in neck disorders affecting postural stability, head and eye movement control. Man Therap. 2008;13(1):2–11. 17702636 10.1016/j.math.2007.06.003

[8] Peng B, Yang L, Li Y, Liu T, Liu Y. Cervical proprioception impairment in neck pain-pathophysiology, clinical evaluation, and management: a narrative review. Pain Ther. 2021;10(1):143–64. 10.1007/s40122-020-00230-z. 33464539 10.1007/s40122-020-00230-z PMC8119582

[9] Li Y, Yang L, Dai C, Peng B. Proprioceptive cervicogenic dizziness: a narrative review of pathogenesis, diagnosis, and treatment. J Clin Med. 2022;11(21):6293. 36362521 10.3390/jcm11216293 PMC9655761

[10] Eva-Maj M, Hans W, Per-Anders F, Mikael K, Måns M. Experimentally induced deep cervical muscle pain distorts head on trunk orientation. Eur J Appl Physiol. 2013;113:2487–99. 23812089 10.1007/s00421-013-2683-y

[11] Kristjansson E, Treleaven J. Sensorimotor function and dizziness in neck pain: implications for assessment and management. J Orthop Sports Phys Therapy. 2009;39(5):364–77. 10.2519/jospt.2009.2834 19411769

[12] Elsig S, Allet L, Bastiaenen CHG, de Bie R, Hilfiker R. Reliability and measurement error of sensorimotor tests in patients with neck pain: a systematic review. Archives Physiotherapy. 2023;13(1):15. 10.1186/s40945-023-00170-9 PMC10428553 37582811

[13] Röijezon U, Jull G, Blandford C, Daniels A, Michaelson P, Karvelis P, et al. Proprioceptive disturbance in chronic neck pain: discriminate validity and reliability of performance of the clinical cervical movement sense test. Front Pain Res. 2022;3:908414. 10.3389/fpain.2022.908414 PMC9299354 35875476

[14] Sarig Bahat H, Watt P, Rhodes M, Hadar D, Treleaven J. High-vs. low-tech cervical movement sense measurement in individuals with neck pain. Musculoskelet Sci Pract. 2020;45:102097. 10.1016/j.msksp.2019.102097. 32056822 10.1016/j.msksp.2019.102097

[15] Werner IM, Ernst MJ, Treleaven J, Crawford RJ. Intra and interrater reliability and clinical feasibility of a simple measure of cervical movement sense in patients with neck pain. BMC Musculoskelet Disord. 2018;19(1):358. 10.1186/s12891-018-2287-0. 30290759 10.1186/s12891-018-2287-0 PMC6173874

[16] Treleaven J, Dillon M, Fitzgerald C, Smith C, Wright B, Sarig-Bahat H. Change in a clinical measure of cervical movement sense following four weeks of kinematic training. Musculoskelet Sci Pract. 2021;51:102312. 10.1016/j.msksp.2020.102312. 33272876 10.1016/j.msksp.2020.102312

[17] Ernst MJ, Williams L, Werner IM, Crawford RJ, Treleaven J. Clinical assessment of cervical movement sense in those with neck pain compared to asymptomatic individuals. Musculoskelet Sci Pract. 2019;43:64–9. 31277033 10.1016/j.msksp.2019.06.006

[18] Schmidt RA, Lee TD, Winstein C, Wulf G, Zelaznik HN. Motor control and learning: a behavioral emphasis. Human kinetics, USA; 2018.

[19] Heitz RP. The speed-accuracy tradeoff: history, physiology, methodology, and behavior. Front NeuroSci. 2014;8:150. 24966810 10.3389/fnins.2014.00150 PMC4052662

[20] Treleaven J. Management of sensorimotor control in musculoskeletal disorders. In: Falla D, Cook C, Lewis J, McCarthy C, Sterling M, editors. *Grieve’s Modern Musculoskel Physiotherapy*. E-Book. 5th ed. London: Elsevier; 2024. p.374-390..

[21] Haavik H, Kumari N, Holt K, Niazi IK, Amjad I, Pujari AN, et al. The contemporary model of vertebral column joint dysfunction and impact of high-velocity, low-amplitude controlled vertebral thrusts on neuromuscular function. Eur J Appl Physiol. 2021;121(10):2675–720. 10.1007/s00421-021-04727-z. 34164712 10.1007/s00421-021-04727-z PMC8416873

[22] Giacalone A, Febbi M, Magnifica F, Ruberti E. The effect of high velocity low amplitude cervical manipulations on the musculoskeletal system: literature review. Cureus. 2020;12(4): e7682. 10.7759/cureus.7682 PMC7228797 32426194

[23] Masaracchio M, Kirker K, States R, Hanney WJ, Liu X, Kolber M. Thoracic spine manipulation for the management of mechanical neck pain: a systematic review and meta-analysis. PLoS ONE. 2019;14(2):e0211877. 10.1371/journal.pone.0211877. 30759118 10.1371/journal.pone.0211877 PMC6373960

[24] Gevers-Montoro C, Provencher B, Descarreaux M, Ortega de Mues A, Piché M. Neurophysiological mechanisms of chiropractic spinal manipulation for spine pain. Eur J Pain. 2021;25(7):1429–48. 33786932 10.1002/ejp.1773

[25] Alanazi MS, Degenhardt B, Kelley-Franklin G, Cox JM, Lipke L, Reed WR. Neuromuscular response to high-velocity, low-amplitude spinal manipulation—an overview. Medicina. 2025;61(2):187. 40005304 10.3390/medicina61020187 PMC11857552

[26] Gyer G, Michael J, Inklebarger J, Tedla JS. Spinal manipulation therapy: is it all about the brain? A current review of the neurophysiological effects of manipulation. J Integr Med. 2019;17(5):328–37. 31105036 10.1016/j.joim.2019.05.004

[27] Reed WR, Long CR, Kawchuk GN, Pickar JG. Neural responses to the mechanical characteristics of high velocity, low amplitude spinal manipulation: effect of specific contact site. Man Therap. 2015;20(6):797–804. 10.1016/j.math.2015.03.008. 25841562 10.1016/j.math.2015.03.008 PMC4584162

[28] Niazi IK, Navid MS, Merkle C, Amjad I, Kumari N, Trager RJ, et al. A randomized controlled trial comparing different sites of high-velocity low amplitude thrust on sensorimotor integration parameters. Sci Rep. 2024;14(1):1159. 38216596 10.1038/s41598-024-51201-9 PMC10786886

[29] Nim C, Aspinall SL, Cook CE, Corrêa LA, Donaldson M, Downie AS, et al. The effectiveness of spinal manipulative therapy in treating spinal pain does not depend on the application procedures: a systematic review and network Meta-analysis. J Orthop Sports Phys Ther. 2025;55(2):109–22. 10.2519/jospt.2025.12707. 39869665 10.2519/jospt.2025.12707

[30] Nim CG, Downie A, O’Neill S, Kawchuk GN, Perle SM, Leboeuf-Yde C. The importance of selecting the correct site to apply spinal manipulation when treating spinal pain: myth or reality? A systematic review. Sci Rep. 2021;11(1):23415. 34862434 10.1038/s41598-021-02882-z PMC8642385

[31] Nim CG, Kawchuk GN, Schiøttz-Christensen B, O’Neill S. The effect on clinical outcomes when targeting spinal manipulation at stiffness or pain sensitivity: a randomized trial. Sci Rep. 2020;10(1):14615. 32884045 10.1038/s41598-020-71557-y PMC7471938

[32] Sørensen PW, Nim CG, Poulsen E, Juhl CB. Spinal manipulative therapy for nonspecific low back pain: does targeting a specific vertebral level make a difference? A systematic review with meta-analysis. J Orthop Sports Phys Therapy. 2023;53(9):529–39. 10.2519/jospt.2023.11962 37506306

[33] Kulkarni V, Chandy M, Babu K. Quantitative study of muscle spindles in suboccipital muscles of human foetuses. Neurol India. 2001;49(4):355. 11799407

[34] Sung YH, Suboccipital Muscles FH, Posture, Dizziness C. Med (Kaunas). 2022;58(12). 10.3390/medicina58121791. 10.3390/medicina58121791 PMC9786116 36556992

[35] Sung Y-H. Upper cervical spine dysfunction and Dizziness. J Exerc Rehabilitation. 2020;16(5):385. 10.12965/jer.2040612.306 PMC7609854 33178639

[36] Treleaven J, Clamaron-Cheers C, Jull G. Does the region of pain influence the presence of sensorimotor disturbances in neck pain disorders? Man Therap. 2011;16(6):636–40. 10.1016/j.math.2011.07.008. 21890397 10.1016/j.math.2011.07.008

[37] Hopewell S, Chan A-W, Collins GS, Hróbjartsson A, Moher D, Schulz KF et al. CONSORT 2025 statement: updated guideline for reporting randomised trials. Lancet. 2025;405(10489):1633–1640. 10.1016/S0140-6736(25)00672-5 40245901

[38] Betsch MW, Blizzard SR, Shinseki MS, Yoo JU. Prevalence of degenerative changes of the atlanto-axial joints. Spine J. 2015;15(2):275–80. 10.1016/j.spinee.2014.09.011. 25277533 10.1016/j.spinee.2014.09.011

[39] Arjona Retamal JJ, Fernández Seijo A, Torres Cintas JD, de-la-Llave-Rincón AI, Caballero Bragado A. Effects of instrumental, manipulative and soft tissue approaches for the suboccipital region in subjects with chronic mechanical neck Pain. A randomized controlled trial. Int J Environ Res Public Health. 2021;18(16). 10.3390/ijerph18168636. 10.3390/ijerph18168636 PMC8392061 34444389

[40] Treede R-D, Rief W, Barke A, Aziz Q, Bennett MI, Benoliel R, et al. Chronic pain as a symptom or a disease: the IASP classification of chronic pain for the international classification of diseases (ICD-11). Pain. 2019;160(1):19–27. 30586067 10.1097/j.pain.0000000000001384

[41] Langridge N, Gale GF. Examination of the upper cervical region. In: Ryder D, Barnard K, editors. Petty’s Musculoskel Examination and assessment.E-Book. 6th ed. London: Elsevier; 2023. p.196 -220.

[42] Gómez F, Escribá P, Oliva-Pascual-Vaca J, Méndez-Sánchez R, Puente-González AS. Immediate and short-term effects of upper cervical high-velocity, low-amplitude manipulation on standing postural control and cervical mobility in chronic nonspecific neck pain: A randomized controlled trial. J Clin Med. 2020;9(8). 10.3390/jcm9082580. 10.3390/jcm9082580 PMC7463842 32784959

[43] Drayer K, Kauwe M. Effects of cervical spine manipulation on balance and joint proprioception in asymptomatic Individuals: Plausibility and Pilot Study. 2013.

[44] Dunning JR, Butts R, Mourad F, Young I, Fernandez-de-las Peñas C, Hagins M, et al. Upper cervical and upper thoracic manipulation versus mobilization and exercise in patients with cervicogenic headache: a multi-center randomized clinical trial. BMC Musculoskelet Disord. 2016;17(1):64. 10.1186/s12891-016-0912-3. 26852024 10.1186/s12891-016-0912-3 PMC4744384

[45] Dunning JR, Cleland JA, Waldrop MA, Arnot C, Young I, Turner M, et al. Upper cervical and upper thoracic thrust manipulation versus nonthrust mobilization in patients with mechanical neck pain: a multicenter randomized clinical trial. J Orthop Sports Phys Therapy. 2012;42(1):5–18. 10.2519/jospt.2012.3894 21979312

[46] Nogueira N, Oliveira-Campelo N, Lopes Â, Torres R, Sousa ASP, Ribeiro F. The acute effects of manual and instrument-assisted cervical spine manipulation on pressure pain threshold, pressure pain perception, and muscle-related variables in asymptomatic subjects: a randomized controlled trial. J Manipulative Physiol Ther. 2020;43(3):179–88. 10.1016/j.jmpt.2019.05.007. 32951766 10.1016/j.jmpt.2019.05.007

[47] Dunning J, Rushton A. The effects of cervical high-velocity low-amplitude thrust manipulation on resting electromyographic activity of the biceps brachii muscle. Man Therap. 2009;14(5):508–13. 10.1016/j.math.2008.09.003. 19027344 10.1016/j.math.2008.09.003

[48] Röijezon U, Faleij R, Karvelis P, Georgoulas G, Nikolakopoulos G. A new clinical test for sensorimotor function of the hand–development and preliminary validation. BMC Musculoskelet Disord. 2017;18:1–11. 28950843 10.1186/s12891-017-1764-1 PMC5615462

[49] Cohen J. Statistical power analysis for the behavioral sciences. 2nd ed. Hillsdale, NJ: Lawrence Erlbaum Associates; 1988

[50] West RM. Best practice in statistics: the use of log transformation. Ann Clin Biochem. 2022;59(3):162–5. 10.1177/00045632211050531. 34666549 10.1177/00045632211050531 PMC9036143

[51] Miranda IF, Facchini D, Manfio EF. Influence of cervical spine manipulation on neck joint position sense error in patients with chronic neck pain. Man Therapy Posturology Rehabilitation J. 2016; 14:405.

[52] Smith DL, Dainoff MJ, Smith JP. The effect of chiropractic adjustments on movement time: a pilot study using Fitts law. J Manip Physiol Ther. 2006;29(4):257–66. 10.1016/j.jmpt.2006.03.009 16690379

[53] Passmore SR, Burke JR, Good C, Lyons JL, Dunn AS. Spinal manipulation impacts cervical spine movement and fitts’ task performance: a single-blind randomized before-after trial. J Manipulative Physiol Ther. 2010;33(3):189–92. 10.1016/j.jmpt.2010.01.007. 20350671 10.1016/j.jmpt.2010.01.007

[54] Gelley GM, Passmore SR, Glazebrook CM, MacNeil BJ. Effect of spinal manipulation on eye and head movement performance in participants with chronic neck pain: an observational study. J Manipul Physiol Ther. 2025;48:1–5. 10.1016/j.jmpt.2025.08.006 41026066

[55] Hage R, Detrembleur C, Dierick F, Brismée JM, Roussel N, Pitance L. Sensorimotor performance in acute-subacute non-specific neck pain: a non-randomized prospective clinical trial with intervention. BMC Musculoskelet Disord. 2021;22(1):1017. 10.1186/s12891-021-04876-4. 34863120 10.1186/s12891-021-04876-4 PMC8645120

[56] Taylor JA, Ivry RB. The role of strategies in motor learning. Ann N Y Acad Sci. 2012;1251:1–12. 10.1111/j.1749-6632.2011.06430.x. 22329960 10.1111/j.1749-6632.2011.06430.x PMC4330992

[57] Mazzoni P, Krakauer JW. An implicit plan overrides an explicit strategy during visuomotor adaptation. J Neurosci. 2006;26(14):3642–5. 16597717 10.1523/JNEUROSCI.5317-05.2006 PMC6674132

[58] Robinault L, Holobar A, Crémoux S, Rashid U, Niazi IK, Holt K, et al. The effects of spinal manipulation on motor unit behavior. Brain Sci. 2021;11(1). 10.3390/brainsci11010105. 10.3390/brainsci11010105 PMC7828823 33466707

[59] Franklin S, Wolpert DM, Franklin DW. Visuomotor feedback gains upregulate during the learning of novel dynamics. J Neurophysiol. 2012;108(2):467–78. 22539828 10.1152/jn.01123.2011 PMC3404796

[60] Calalo JA, Roth AM, Lokesh R, Sullivan SR, Wong JD, Semrau JA, et al. The sensorimotor system modulates muscular co-contraction relative to visuomotor feedback responses to regulate movement variability. J Neurophysiol. 2023;129(4):751–66. 10.1152/jn.00472.2022. 36883741 10.1152/jn.00472.2022 PMC10069957

[61] Dhawale AK, Smith MA, Ölveczky BP. The role of variability in motor learning. Annu Rev Neurosci. 2017;40:479–98. 28489490 10.1146/annurev-neuro-072116-031548 PMC6091866

[62] Miyamoto YR, Wang S, Smith MA. Implicit adaptation compensates for erratic explicit strategy in human motor learning. Nat Neurosci. 2020;23(3):443–55. 10.1038/s41593-020-0600-3. 32112061 10.1038/s41593-020-0600-3

[63] Wu HG, Miyamoto YR, Castro LNG, Ölveczky BP, Smith MA. Temporal structure of motor variability is dynamically regulated and predicts motor learning ability. Nat Neurosci. 2014;17(2):312–21. 24413700 10.1038/nn.3616 PMC4442489

[64] Gevers-Montoro C, Provencher B, Descarreaux M, Ortega de Mues A, Piché M. Clinical effectiveness and efficacy of chiropractic spinal manipulation for spine pain. Front Pain Res (Lausanne). 2021;2:765921. 10.3389/fpain.2021.765921. 35295422 10.3389/fpain.2021.765921 PMC8915715

[65] Bialosky JE, Bishop MD, George SZ, Robinson ME. Placebo response to manual therapy: something out of nothing? J Man Manip Ther. 2011;19(1):11–9. 10.1179/2042618610y.0000000001. 22294849 10.1179/2042618610Y.0000000001 PMC3172952

[66] Bexander CS, Mellor R, Hodges PW. Effect of gaze direction on neck muscle activity during cervical rotation. Exp Brain Res. 2005;167:422–32. 16193272 10.1007/s00221-005-0048-4

