# Supplementary material for: Immediate effects of upper cervical spinal manipulation on cervical sensorimotor control in individuals with chronic primary cervical pain: an exploratory randomized controlled trial
Source: Chiropr Man Therap. 2026 Jan 16;34:4. doi: 10.1186/s12998-026-00626-2 (PMC12892738; doi:10.1186/s12998-026-00626-2)
Supplement: Supplementary file 1 — Supplementary Material 1 [file 12998_2026_626_MOESM1_ESM.pdf]

# Customized Algorithm for Video Analysis in MATLAB

## - Cervical Movement Sense (CMS) Test in the Zigzag Pattern

Minwoo Lee & Yongwoo Lee

## Overview

This customized algorithm was developed in MATLAB software (version R2023a; The MathWorks Inc., USA), based on the methodology described by Röijezon et al. [1, 2]. Although the original source code was not utilized, the algorithm and procedures were independently implemented using similar image processing techniques reported in previous studies [1, 2], and were executed with functions from the *Image Processing Toolbox*. Whereas the original approach fully automated all processing steps, the present method was modified to allow manual selection of the start and end frames, thereby enhancing precision and user control.

**Step 1.** Extracting the Black Line and Detecting the Four Reference Corners in the Zigzag Pattern

The recorded video file (.mp4) was imported, and the initial frame was selected as a reference image to minimize noise from unexpected variations in lighting, shaking, or vibrations during video recording. This frame was converted to grayscale, and global thresholding for image binarization was applied to extract the black line in the zigzag pattern. The coordinates of four reference corners (upper left, upper right, lower left, and lower right) were manually marked in this frame as directional references to enable automatic detection with improved precision and consistency. As the laser dot always originated from one of these corners, a single start point was also manually marked in this frame and then matched to the nearest automatically detected corner (**Figure 1**). At this stage, the scale factor (mm per pixel) was calculated using the Euclidean distance between the upper left and upper right reference corners, based on the known physical length of the horizontal segment (234 mm).

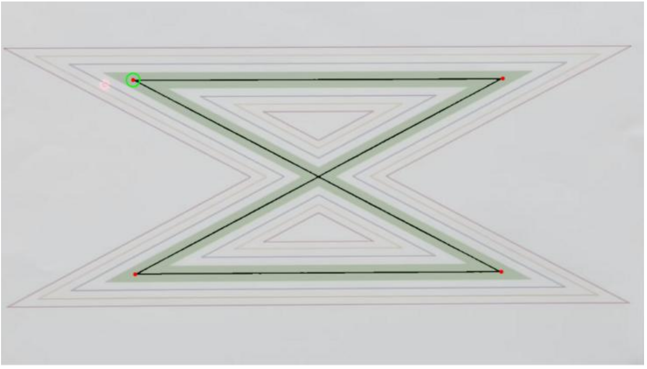

**Figure 1.** The zigzag pattern, extracted as a black line using global thresholding for image binarization, was overlaid on the original RGB image (initial frame). The four reference corners (upper left, upper right, lower left, and lower right) were marked with red dots, and the start point (located at the upper left corner in this figure) was indicated by a green circle. All points were automatically detected based on manually marked locations in the initial frame.

**Step 2.** Extracting the Laser Dot and Normalizing Its Size

Each frame of the recorded video file (.mp4) was imported and converted to grayscale by extracting the red (R) channel. A global threshold was then applied to extract the laser dot through image binarization (**Figure 2**). Due to the characteristics of image processing, the laser dot in each frame was not a perfect circle or ellipse, and its size varied across frames. Therefore, normalization of the laser dot size was performed to account for both intra- and inter-frame variability. For intra-frame analysis, the `regionprops` function in MATLAB software was used to calculate the `Centroid` (center of the dot), `MajorAxisLength`, `MinorAxisLength`, and `Area` of the laser dot in each frame. The average of the `MajorAxisLength` and `MinorAxisLength` was defined as the diameter of the laser dot for that frame. For inter-frame analysis, excluding frames in which no laser dot was detected, the average laser dot diameter across all frames was used as the reference for normalization. Finally, in each frame, the laser dot was adjusted to a perfect circle using its centroid, the normalized diameter, and the corresponding normalized radius.

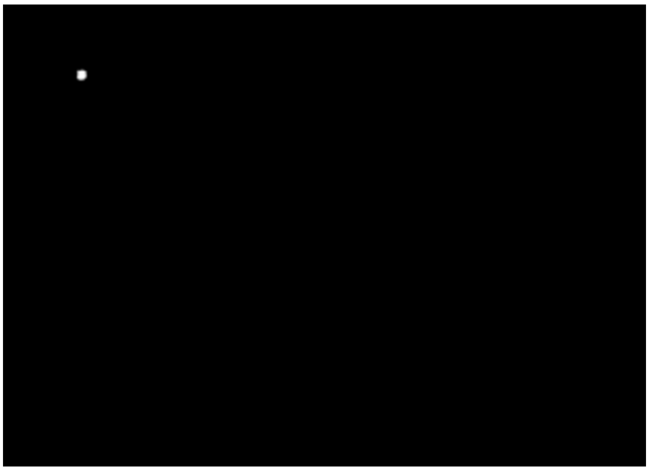

**Figure 2.** The laser dot was extracted through image binarization by applying a global threshold.

**Step 3.** Calculating and Preprocessing the Frame-by-Frame Pixel Distance from the Start Point

The Euclidean pixel distance between the laser dot and the start point was calculated for each frame. Pixel distances in frames in which the laser dot was not detected were left as missing values. Outliers were identified based on deviations from the centered moving average relative to the moving standard deviation. Identified outliers were imputed with the simple average of the nearest valid previous and subsequent distances, while missing values were left unchanged.

**Step 4.** Visualizing the Pixel Distance and Manually Selecting the Start and End Frames of Laser Dot Movement

The frame-by-frame pixel distance plot was generated to visualize the positional change of the laser dot relative to the start point. In this plot, the x-axis represents the frame number, and the y-axis represents the pixel distance from the start point. To enable more precise analysis and provide user control, the start and end frames for calculating the CMS kinematic variables were manually selected. In the present study, participants were instructed to hold the laser dot stationary at the start point for approximately one second before initiating the movement, and to do the same upon returning to the start point at the end of the trial. The start frame was identified as the frame showing the minimum pixel distance near the onset of the movement and a sharply increasing slope. The end frame was defined as

the first frame exhibiting the minimum pixel distance and a near-zero slope, indicating the termination of the laser dot movement (**Figure 3**).

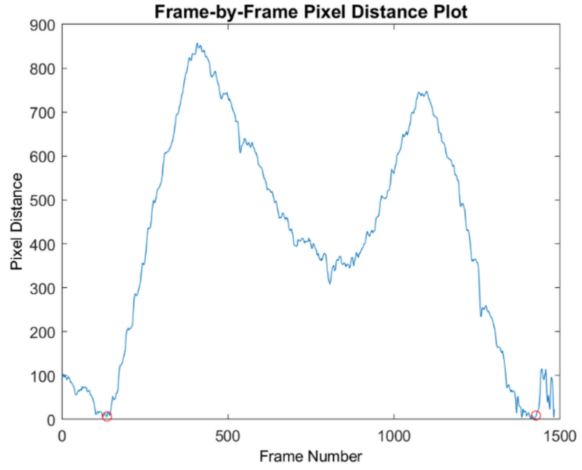


**Figure 3.** Frame-by-frame pixel distance plot. The start and end frames were manually selected.

**Step 5.** Determining Laser Dot Contact with the Black Line

To determine whether the laser dot was in contact with the black line between the start and end frames, the shortest Euclidean distance from the centroid of the laser dot to each of the four line segments was calculated using a `GetPointSegmentDistance` (custom point-to-segment distance function) based on dot product and vector projection. The minimum of these four distances was then compared against the sum of the laser dot radius and half of the black line thickness in pixels (converted using the scale factor calculated in **Step 1**). If the minimum distance was less than or equal to this value, the laser dot was considered to be in contact with the black line. Otherwise, it was considered not in contact. This binary outcome (1 = contact, 0 = no contact) was recorded for each frame (**Figure 4**).

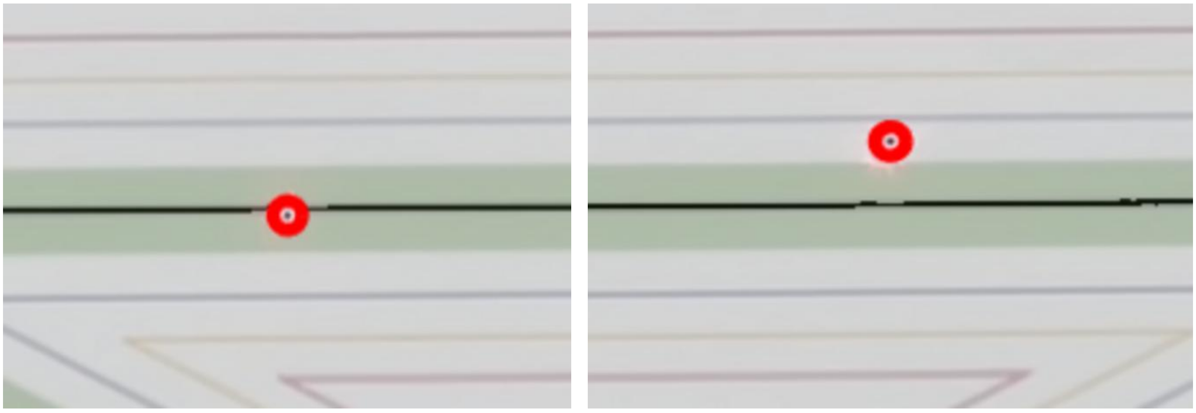

(a) (b)

**Figure 4.** Determining laser dot contact with the black line. The laser dot is visualized using its normalized diameter and radius. (a) Contact, (b) No contact.

**Step 6.** Calculating the CMS Kinematic Variables

Movement time (MT) was calculated as the time interval between the manually selected start

and end frames. Movement speed (MS) was calculated as the cumulative distance (sum of inter-frame displacements between the start and end frames) divided by MT and expressed in mm/s using the scale factor. Pixel distances from frames in which the laser dot was not detected were treated as missing values and excluded from the MS calculation. Movement accuracy (MA) was calculated as the percentage of frames in which the laser dot contacted the black line. Movement accuracy-time ratio (MAT) was calculated by dividing MA by MT.

**Implementation Considerations and Limitations**

- The performance of binarization for the zigzag pattern and laser dot may vary depending on the laboratory environment and camera conditions. For this reason, it is necessary to appropriately tune the `zz_threshold` (for extracting the black line in the zigzag pattern) and `laser_threshold` (for extracting the laser dot) parameters to ensure optimal performance.
- This customized algorithm was developed based on the use of a red laser dot in the CMS test, where the red (R) channel from the RGB video frames was extracted and binarized using the `laser_threshold` parameter. If a green or other colored laser dot was used, the corresponding channel (e.g., G channel for green, B channel for blue) must be extracted, and the `laser_threshold` parameter re-adjusted to ensure optimal detection.
- To enable automatic detection of the four reference corners, manual marking must be performed carefully within the allowable search region. Accordingly, it is necessary to appropriately tune the `search_box_size` parameter, which defines the pixel range for detecting the four reference corners accurately.
- Outliers in the frame-by-frame pixel distance may vary depending on the laboratory environment and camera conditions. It is necessary to appropriately tune the `window_size` and `std_threshold` parameters for reliable detection and correction.
- Significant deviations from the predefined movement path or sequence of the laser dot may lead to unintended incorrect results when determining contact with the black line, as the closest line segment among the four may be incorrectly identified. Therefore, it is necessary to ensure that the laser dot precisely follows its predefined movement path and sequence.

**Reference**

1. Röijezon U, Jull G, Blandford C, Daniels A, Michaelson P, Karvelis P, et al. Proprioceptive disturbance in chronic neck pain: Discriminate validity and reliability of performance of the clinical cervical movement sense test. Front Pain Res (Lausanne). 2022;3:908414.
2. Röijezon U, Faleij R, Karvelis P, Georgoulas G, Nikolakopoulos G. A new clinical test for sensorimotor function of the hand–development and preliminary validation. BMC Musculoskelet Disord. 2017;18:1-11.
